# Fine-scale population genetic structure of dengue mosquito vector, *Aedes aegypti*, in Metropolitan Manila, Philippines

**DOI:** 10.1371/journal.pntd.0008279

**Published:** 2020-05-04

**Authors:** Thaddeus M. Carvajal, Kohei Ogishi, Sakiko Yaegeshi, Lara Fides T. Hernandez, Katherine M. Viacrusis, Howell T. Ho, Divina M. Amalin, Kozo Watanabe

**Affiliations:** 1 Center for Marine Environmental Studies (CMES)–Ehime University, Matsuyama, Japan; 2 Department of Civil and Environmental Engineering—Ehime University, Matsuyama, Japan; 3 Biology Department–De La Salle University, Taft Ave Manila, Philippines; 4 Biological Control Research Unit, Center for Natural Science and Environmental Research—De La Salle University, Taft Ave Manila, Philippines; 5 Department of Civil and Environmental Engineering, University of Yamanashi, Kofu, Japan; 6 Department of Biological Sciences, Trinity University of Asia, Quezon City, Philippines; Faculty of Science, Mahidol University, THAILAND

## Abstract

Dengue is a highly endemic disease in Southeast Asia and is transmitted primarily by the mosquito, *Aedes aegypti*. The National Capital Region (NCR) of the Philippines, or Metropolitan Manila, is a highly urbanized area that is greatly affected by this arboviral disease. Urbanization has been shown to increase the dispersal of this mosquito vector. For this reason, we conducted a fine-scale population genetic study of *Ae*. *aegypti* in this region. We collected adult *Ae*. *aegypti* mosquitoes (n = 526 individuals) within the region (n = 21 study areas) and characterized the present population structure and the genetic relatedness among mosquito populations. We genotyped 11 microsatellite loci from all sampled mosquito individuals and analyzed their genetic diversity, differentiation and structure. The results revealed low genetic differentiation across mosquito populations which suggest high gene flow and/or weak genetic drift among mosquito populations. Bayesian analysis indicated multiple genetic structures (K = 3–6), with no clear genetically distinct population structures. This result implies the passive or long-distance dispersal capability nature *Ae*. *aegypti* possibly through human-mediated transportation. The constructed dendrogram in this study describes the potential passive dispersal patterns across Metropolitan Manila. Furthermore, spatial autocorrelation analysis showed the limited and active dispersal capability (<1km) of the mosquito vector. Our findings are consistent with previous studies that investigated the genetic structure and dual (active and passive) dispersal capability of *Ae*. *aegypti* in a fine-scale highly urbanized area.

## Author summary

*Aedes aegypti* is an efficient vector of dengue due to its highly adaptive nature to the urban environment. Although it is observed to have a short dispersal (active) capability, it has been shown to be capable of traveling long distances (passive) via human-mediated transportation. This duality may expand the distribution of the mosquito vector in urbanized areas. In this study, we examined the population genetic structure of *Ae*. *aegypti* in a highly urbanized and dengue-endemic region of the Philippines, Metropolitan Manila. Our findings indicated the dual dispersal nature of *Ae*. *aegypti*. The use of microsatellites as genetic markers also allowed us to describe the potential long-distance dispersal patterns, possibly through human-aided land transportation via the existing road networks of Metropolitan Manila.

## Introduction

Dengue disease is the most prevalent mosquito-borne viral infection in tropical and subtropical countries [1] with approximately 2.5 billion people at risk of contracting the disease worldwide [2]. The Philippines considers dengue as a major public health concern and has been ranked fourth in Southeast Asia with the highest number of dengue cases reported annually [3]. In particular, the National Capital Region (NCR), also known as Metropolitan Manila, is one of the main areas affected by the disease and an average of 16% of the total number of cases reported in the Philippines from 2009 to 2014 [4]. Interestingly, the first recorded dengue epidemic in Southeast Asia occurred in this region in 1954 [5]. Although a dengue vaccine is available [6], the World Health Organization [2] still recommends disease prevention and control towards the mosquito vector.

The transmission of this arboviral disease is through the bite of either the *Aedes aegypti* or *Ae*. *albopictus* mosquito vector. The former is identified as the primary vector of the disease, and as such, its biology has been extensively studied including its reproduction and development [7–9], survivability [10–11], dispersal [12–14], and ecology[15–16]. In many population genetic studies of *Ae*. *aegypti*, microsatellites have been used as a molecular marker of choice for differentiating macro- [17,19] and micro-geographic [20,21,22] scale mosquito populations due to its high polymorphism, co-dominance, and broad genome distribution [23]. Molecular genotyping of the mosquito vector using these markers has shown additional insights towards the microevolution of the mosquito vector [17–19], revealing the population’s gene flow pattern which can be interpreted as the mosquito vector’s dispersal ability [20,24].

Urbanization has been a key factor in the widespread occurrence of dengue disease worldwide and has contributed to the dispersal of the mosquito vector [16]. *Ae*. *aegypti* has been documented to thrive in highly urbanized areas, creating the ideal condition for a dengue epidemic [25,26]. As such, investigating the impact of urbanization on *Ae*. *aegypti*’s population genetic structure has sparked research interest, particularly regarding its micro-evolutionary process [24, 26]. A growing urban area and human population density can affect the mosquito populations on a genetic level. An increase in urbanization can reduce genetic diversity and promote homogenization of the genetic structure of mosquito populations. However, when an area reaches complete and/or stable urbanization, the mosquito populations may reach peak abundance, allowing an increase in genetic diversity and thereby, establishing heterogenous genetic structures of mosquito populations [22]; a phenomenon that has been observed in urbanized cities in Brazil [22], Thailand [21], Indonesia [27], Cambodia and Myanmar [19].

Although dengue is endemic in the Philippines, there are a limited number of studies that investigate the population genetics of *Ae*. *aegypti* in the country. These studies revealed the seasonal genetic structuring and expansion of the mosquito vector [28] and the connectivity among its populations from different island seaports [29], but provide little insight into genetic structuring of *Ae*. *aegypti* in highly urbanized and dengue-endemic regions in the country. In this study, we selected Metropolitan Manila because it represents an ideal setting to investigate how urbanization can shape the present genetic structure of *Ae*. *aegypti*. Previous studies conducted in this area have only investigated the association between dengue epidemiology and climate [5,30,31] and the population structure using wing geometric morphometrics [32], offering little information about its genetic structure and relatedness. Therefore, the study collected adult *Ae*. *aegypti* samples from several sites within Metropolitan Manila in order to characterize the population genetic structure and relatedness among mosquito populations.

## Methods

### Study area and mosquito sampling

Metropolitan Manila, a highly urbanized area, is the National Capital Region (NCR) of the Philippines with an area of 636 km^2^. It is located at the eastern shore of Manila Bay in Southwestern Luzon (14°50′ N Latitude, 121°E Longitude). It is composed of 16 cities and 1 municipality with a total population of 12,877,253 [33]. This area is the most urbanized region in the Philippines and is the center of the national government, economy, education and culture, business, manufacturing and importation and exportation [34].

**Fig 1:**
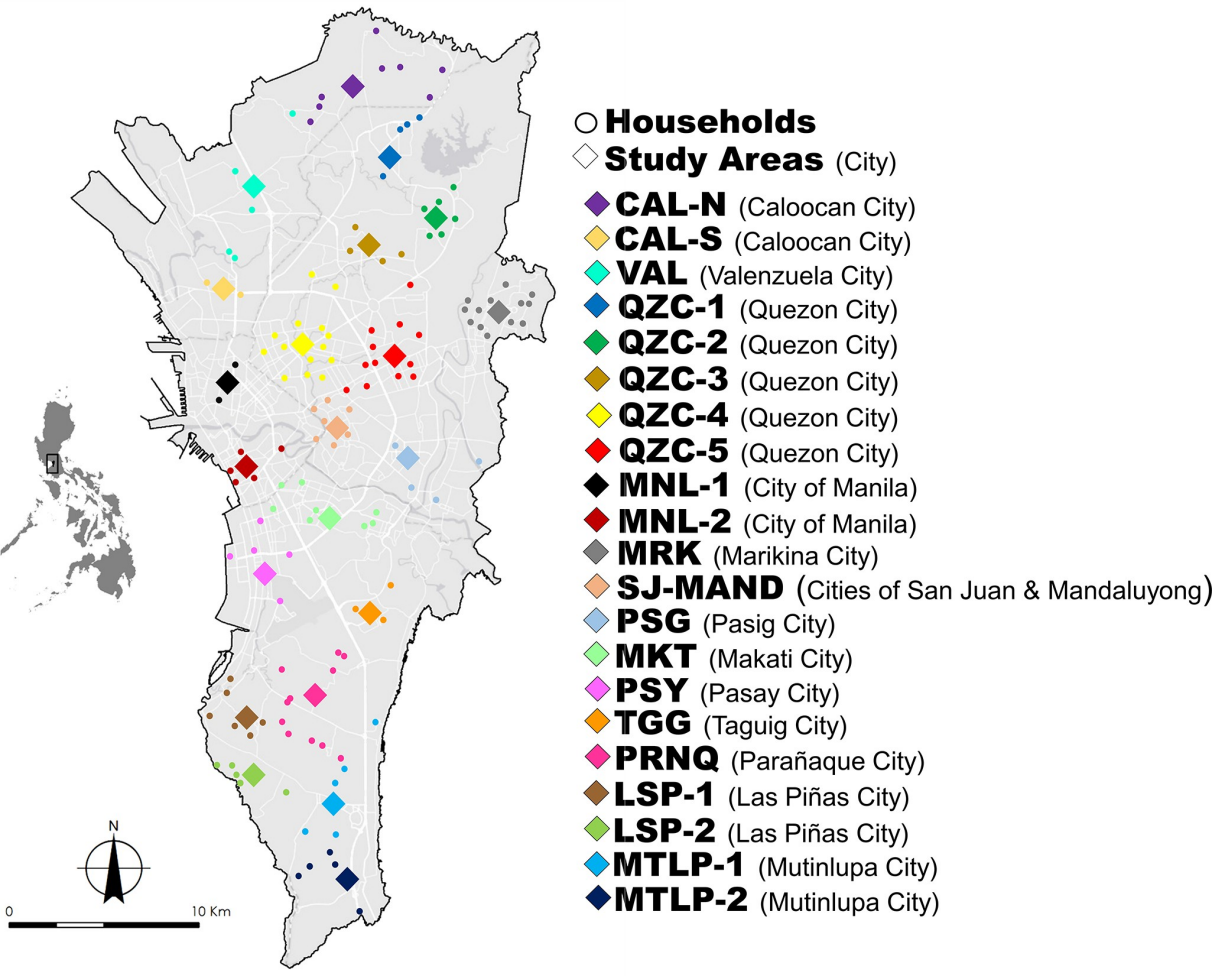
Geographic midpoints of *Ae*. *aegypti* study areas (colored diamond) with their corresponding household sites (colored circles) in Metropolitan Manila. Details of each study area can be seen in S1 Table. The map was prepared using ArcGIS version 10.2.2 from LandsatLook Viewer (http://landsatlook.usgs.gov/).

In this study, Metropolitan Manila was divided into 21 study areas (Fig 1 and S1 Table). Initially, study areas were delineated per city with at least one study area per city. However, some cities comprised either a large or small land sized area, thus designating more study areas. For example, the largest city, in the region Quezon City was divided into five study areas based on its district boundaries. In order to standardize the land size covered by each study area, we merged two small neighboring cities, San Juan and Mandaluyong. All maps were prepared in ArcGIS software version 10.2.2 (ESRI, NC, USA) for further analysis [35].

Households on each study area were selected based on voluntary informed consent for collecting adult *Ae*. *aegypti* mosquitoes inside their premises. The number of households per study area ranged from 2–14 with an average of six households in each area and a total of 151 households. The maximum distance between households within study areas ranged from 1.39–6.17 km. Since the households were widely dispersed across each study area, we calculated the geographical midpoint [36] to assign a single georeferenced location for each study area in the subsequent genetic analysis. The distance among study areas (midpoints) ranged from 2.85–39.66 km.

The majority of the previous population genetic studies of *Ae*. *aegypti* have either used only larval or reared larval-to-adult samples which could lead to potential bias in estimating population genetic parameters due to the sampling of full sibling mosquito larvae [37]. This was evidenced in a population genetic structure study of amphibian larval samples that led to inaccurate estimates of differentiation among populations when compared to adult samples [38,39]. As such, this study targets adult *Ae*. *aegypti* samples rather than conventional egg, or larval samples to prevent collecting mosquito full siblings. Collection of *Ae*. *aegypti* mosquitoes was done by installing a commercially available mosquito UV Light Trap (MosquitoTrap, Jocanima Corporation, Las Pinas City, Philippines) in each household’s outdoor premises for 3–5 days. Collected adult mosquito individuals were sorted, then identified accordingly based on the pictorial keys of Rueda et al. [40] and preserved in 99% ethanol. From May 2014 until January 2015, a total of 526 adult *Ae*. *aegypti* were collected, ranging from 10 to 42 individuals per study area (S1 Table).

### DNA extraction and microsatellite genotyping

The total genomic DNA of individual mosquito sample was extracted using the QIAGEN Blood and Tissue DNEasy Kit (Qiagen, Hilden, Germany) following the modified protocol of Crane [41]. The average DNA concentration per individual mosquito sample was measured at 41.68 ± 34.50 ng/ μL using NanoDrop2000 Spectrophotometer (Thermo Scientific Inc., Waltham, Massachusetts, USA). We selected 11 microsatellite markers out of the 24 loci as listed by Slotman et al. [42] and Chambers et al. [23] by conducting trial runs (PCR and gel electrophoresis) on a subset of *Ae*. *aegypti* samples (n = 8). The selection of the markers was based on the reliability of the amplified products in all samples from different trial runs. The selected markers used in this study were congruent with previous population studies conducted in Southeast Asia [19,21]. The loci were grouped into four sets for multiplex PCR (S2 Table). Each set consisted of fluorescent labeled forward primers with different annealing temperatures during the amplification process. Each set was composed of 1.2 μL of 10X buffer (TaKaRa, Shiga, Japan), 0.8 μL of 25 mM MgCl_2_, 1.6 μL of 10 mM of each dNTP, 0.6 μL of 10 μM forward and reverse primers and 0.08 μL 5.0U/ μL of Taq DNA polymerase (TaKaRa), 1.5 μL of 10% dimethyl sulfoxide (DMSO) (Sigma-Aldrich, St. Louis, Missouri, USA) and 1 μL of template DNA with a final volume of 10 μL. Thermocycling conditions were as follows: initial denaturation at 94°C for 5 minutes, denaturation at 94°C for 30 seconds, annealing (see S2 Table for temperatures and durations for each primer set) extension at 72°C for 30 seconds (35 cycles) and a final incubation step of 72°C for 5 minutes. PCR amplicons were checked by electrophoresis in 3% agarose gels and visualized under UV light using the Chemidoc XRS Chemiluminescent Gel Documentation Cabinet (BIO-RAD). Prior to fragment size analyses, multiplex PCR products were diluted in 1/15 water and then pooled together. One microliter of each diluted pool was added with 0.5 μL of GS 500 Liz Internal Size Standard (Applied Biosystems, USA) and HD formamide for a total volume of 20 μL. Fragment analysis of the amplified products were done using ABI 3500 Genetic Analyzer (Life Technologies) while genotyping was conducted in GeneMapper (Applied Biosystems). Microsatellite data were checked for error, and the presence of null alleles was confirmed with MicroChecker [43]. The dataset was deposited and made publicly available at *vectorbase*.*org* [44] with the population biology project ID: VBP0000554.

The exact Hardy-Weinberg equilibrium (HWE) test and estimations of the linkage disequilibrium (LD) among all pairs of loci were conducted using GENEPOP v4.2.1 [45,46]. Significance levels for multiple testing were corrected using the Bonferroni procedure. Measures of genetic diversity, including mean number of different alleles (*MNa*), mean number of effective alleles (*MNe*), allelic richness (*AR*), private allelic richness (*PAR*), observed heterozygosity (*Ho*), expected heterozygosity (*He*), and inbreeding coefficients (Fis) were estimated using Genetic Analysis in Excel (GenAlEx) version 6.3 [47]. To assess the magnitude of genetic differentiation among sites, uncorrected and corrected pairwise F_ST_ values were calculated using Arlequin v3.5.1.3 [48] and FreeNA [49], respectively, with 10,000 permutations. The analysis of molecular variance (AMOVA) was performed using GenAlEx version 6.3 [47]. Since the corrected pairwise F_ST_ values were calculated by FreeNA [49], we manually calculated the number of pairwise effective migrants using the formula of Slatkin and Barton [50] in GenAlEx version 6.3 [47]. FreeNA [49] was also used to calculate the Cavalli Sforza and Edwards distance. A dendrogram was constructed based on the Cavalli Sforza and Edwards distance using the unweighted pair group with arithmetic mean (UPGMA) in the *fastcluster* package [51] and the optimal number of clusters were determined using the cindex index in the *NbClust* package [52] of the R program [53].

### Genetic structure

The number of genetic groups (K) was inferred using the Bayesian approach in the software Structure version 2.3.2 [54]. The study used admixture models where the alpha value may vary, and independent allele frequencies set at lambda equals to one. Twenty independent runs were performed for each value of K (1–20) with a burn-in phase of 200,000 iterations followed by 600,000 replications. To determine the most likely number of clusters, we calculated the commonly used ΔK statistic as developed by Evanno et al (2005) [55] using the web application Structure Harvester version 0.6.93 [56]. To avoid the effects of uneven sampling [57,58] in the Bayesian analysis using the software Structure version 2.3.2 [54], we standardized the number of individuals per study area to 10 and created five different datasets. These datasets were subjected to Bayesian analysis using the same parameters and analyzed using the Evanno et al (2005) [55] method. The ‘*pophelper’* package [59] in R [53] was used to summarize and visualize the bar plots for the best K statistic identified for each dataset.

### Isolation by distance and spatial autocorrelation

Pairwise geographic distances (km) among study areas and households of the mosquito populations were calculated using Vincenty’s formulae [60] in Microsoft Excel 2016. To test isolation by distance (IBD), pairwise *F*_*ST*_ and geographical distance (km) were examined using Mantel’s test of correlation with 10,000 permutations. Spatial autocorrelation was performed using pairwise Provesti’s genetic distance among mosquito individuals and geographic distance (km) among households with 10,000 permutations and bootstrap replications. Pairwise Provesti’s genetic distance among mosquito individuals was calculated using the ‘*poppr’* package [61] in R [53]. Results of the permutation were considered significant at the 5% level. In this analysis, a correlogram was produced with 45 distance classes at 1 km intervals. Both analyses yielded a correlation coefficient of the two data matrices ranging from –1 to +1, with a test for a significant relationship by random permutation. All analyses were performed using GenAlEx version 6.3 [47].

## Results

### Marker assessment

We observed a total of 113 alleles across 11 microsatellite loci in 21 mosquito populations from Metropolitan Manila (S3 Table). The number of alleles per loci ranged from 3 (F06) to 19 (B07) with an average of 10.25 alleles per loci, suggesting the high polymorphic nature of the selected microsatellites. A total of 91 out of 231 (39%) deviated significantly from the Hardy-Weinberg Equilibrium expectations after sequential Bonferroni correction. Seventy-two of these significant deviations indicated He > Ho, suggesting heterozygosity deficiency (S4 Table). Null alleles were present in four loci (M313, AC4, AG7 and H08) and the null allele frequency ranges from 0.00–0.33 in all loci (S5 Table). The LD test showed a total of 131 out of 1155 (11.30%) pairs of loci with significant LD after Bonferroni corrections, with no locus pair consistently correlated across all mosquito populations (S6 Table).

### Genetic diversity and differentiation

Table 1 shows a summary of the genetic diversity of each mosquito population. The mean number of different alleles ranged from 3.82 (TGG) to 6.36 (QZC-3) while the mean number of effective alleles areas ranged from 2.74 (QZC-1) to 3.51 (LSP-2). On the other hand, the mean allelic richness ranged from 3.24 (QZC-1) to 3.85 (MRK) while the proportion of private alleles ranged from 0.02 (LSP-2) to 0.23 (PSY). All mosquito populations except for one (MKT) did not conform to Hardy-Weinberg equilibrium expectations (He > Ho), indicating heterozygosity deficits and the possibility of inbreeding within each mosquito population.

**Table 1:** Genetic diversity of 21 *Ae*. *aegypti* populations based on 11 microsatellites in Metropolitan Manila, Philippines.

*Study Areas*	*MNa*	*MNe*	*AR*	*PAR*	*Ho*	*He*	*FIS*
CAL-N	5.91	3.09	3.68	0.08	0.493	0.576	0.169
CAL-S	4.73	2.93	3.49	0.11	0.468	0.589	0.215
VAL	4.27	2.93	3.49	0.04	0.508	0.567	0.143
QZC-1	4.45	2.74	3.24	0.10	0.470	0.534	0.111
QZC-2	5.82	2.94	3.45	0.06	0.520	0.545	0.047
QZC-3	6.36	2.92	3.44	0.03	0.525	0.565	0.071
QZC-4	6.00	3.20	3.63	0.08	0.541	0.574	0.077
QZC-5	5.55	3.18	3.37	0.05	0.500	0.565	0.117
MNL-1	6.09	3.30	3.53	0.08	0.524	0.581	0.090
MNL-2	5.36	2.75	3.55	0.03	0.468	0.530	0.132
MRK	5.64	3.09	3.85	0.07	0.511	0.579	0.112
SJ-MND	5.82	3.12	3.29	0.03	0.496	0.574	0.191
PSG	4.91	2.88	3.56	0.08	0.524	0.573	0.099
MKT	5.18	3.00	3.59	0.06	0.558	0.551	0.036
PSY	5.64	2.94	3.71	0.23	0.506	0.551	0.080
TGG	3.82	2.79	3.27	0.05	0.536	0.567	0.071
PRNQ	5.64	2.90	3.78	0.04	0.564	0.580	0.076
LSP-1	5.82	3.13	3.65	0.12	0.536	0.578	0.104
LSP-2	5.00	3.51	3.44	0.02	0.432	0.589	0.296
MTLP-1	5.73	3.08	3.79	0.13	0.438	0.580	0.240
MTLP-2	6.00	3.02	3.51	0.03	0.449	0.577	0.240

*MNa* = mean number of different alleles; *MNe* = mean number of effective alleles; *AR =* allelic richness; *PAR =* private allelic richness; *Ho* = observed heterozygosity; *He =* expected heterozygosity; *FIS =* inbreeding coefficient

The global *F*_ST_ was estimated to be 0.013 where pairwise corrected *F*_ST_ values among mosquito populations ranged from -0.009 to 0.064 (S7 Table). Among these comparisons, 87 out of 201 (41.4%) uncorrected pairwise *F*_ST_ values showed significant genetic differences (S7 Table). The effective number of migrants among mosquito populations ranged from 3.67 to 428.57 migrants (S8 Table) with 13 pairwise estimates were not calculated due to the negative or zero *F*_ST_ values. The results from the AMOVA showed that the *F*_IS_ and *F*_IT_ values were 0.131 and 0.137, respectively (both P < 0.001) (S9 Table), indicating that genetic differences exist among and within individuals.

**Fig 2:**
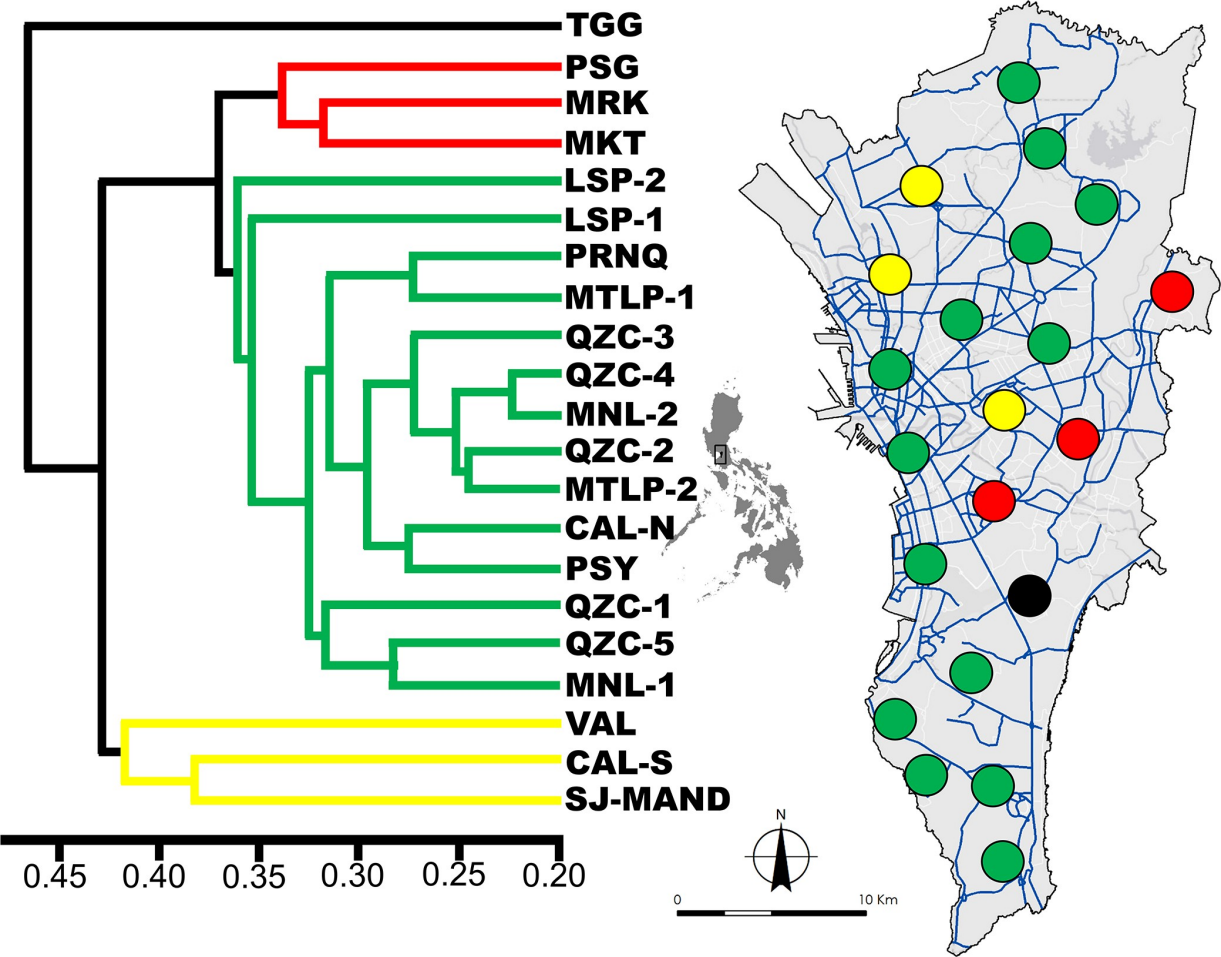
Dendrogram showing the genetic relatedness of each study area based on Cavalli Sforza and Edwards distance. Colored lines indicate the genetic groups (n = 4). The map showing the study areas in respect to their genetic group assignment (colored circles). Blue lines in the map show the primary road network of the region. The map was prepared using ArcGIS version 10.2.2 from LandsatLook Viewer (http://landsatlook.usgs.gov/).

The dendrogram based on Cavalli Sforza and Edwards distance revealed the spatial pattern and distribution of genetically similar mosquito populations (Fig 2). The “cindex” index in NbClust determined the optimal number of cluster groups to be four. One group (green line/circles, Fig 2) had 14 mosquito populations that were highly connected or genetically similar. An extended pattern can be observed from the north down to the southern parts of Metropolitan Manila.

The Mantel test between the pairwise genetic (*F*_ST_) and geographic distances of all mosquito populations showed very low and non-significant correlation (R^2^ = 0.006, p = 0.25), indicating no isolation by distance (S1 Fig). High genetic similarity among adult mosquito individuals was only found up to 1 km based on the spatial autocorrelation analysis (S2 Fig), suggesting limited dispersal capability for *Ae*. *aegypti*.

### Genetic structure

**Fig 3:**
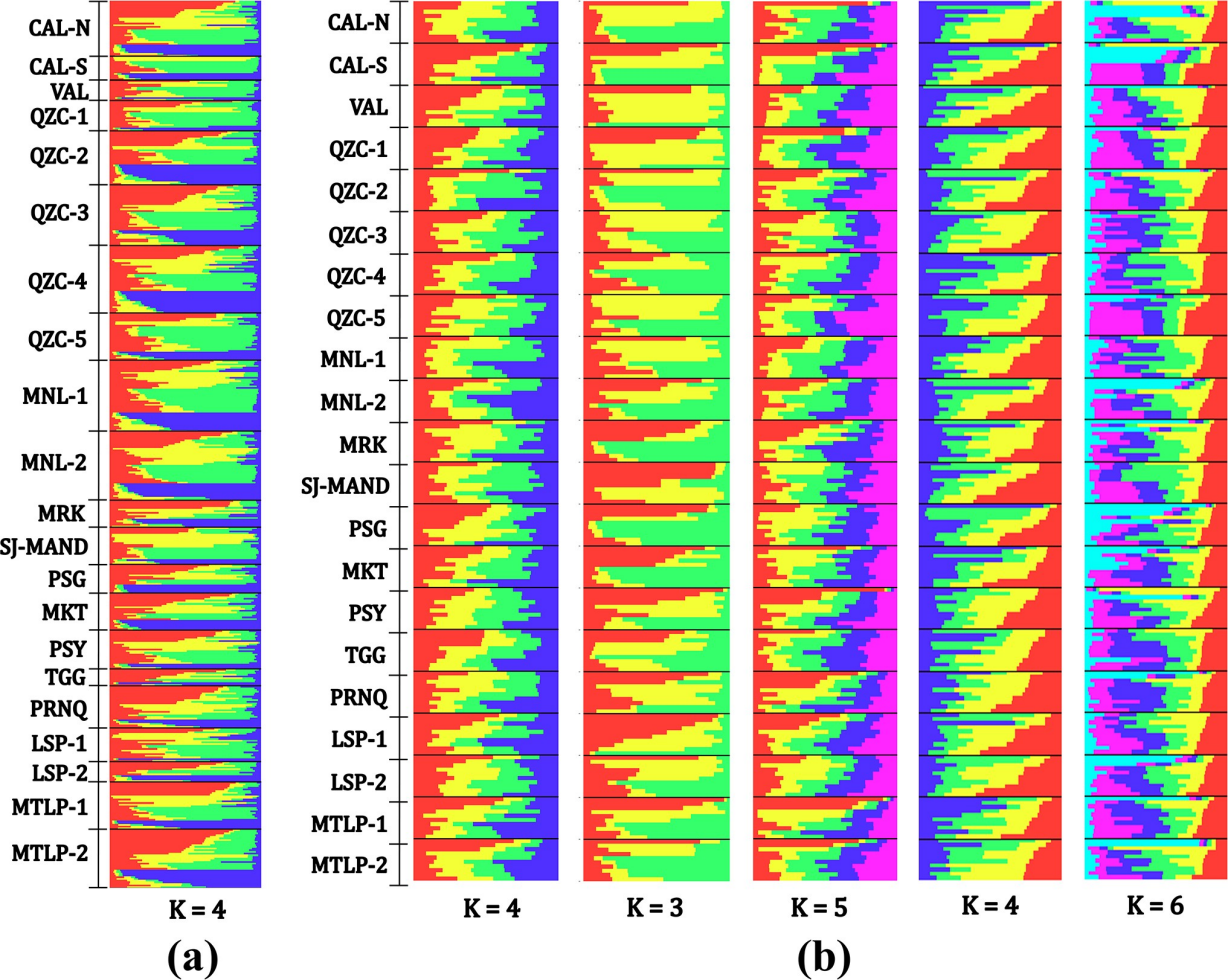
**Individual barplots from the Bayesian analysis using Structure software indicated the distribution of genetic clusters across mosquito populations using (a) all (n = 526) sample individuals and (b) five standardized datasets (n = 10 per study area).** Each individual is represented by a single horizontal line. Brackets are shown to separate study areas. The most likely number of genetic clusters in (a) is K = 4 while, in (b) the number ranges from K = 3 to K = 6.

According to Evanno et al.’s method (2005) [55], the most probable number of genetically differentiated groups using all individuals across mosquito populations is K = 4 (Fig 3, S3 Fig). Furthermore, the standardized datasets showed the most probably numbers of K ranged from 3 to 6 (Fig 3, S3 Fig). All barplots demonstrated no clear genetically-distinct clusters, suggesting high admixture among all mosquito populations.

## Discussion

Our study revealed low genetic differentiation (*F*_ST_) among mosquito populations across Metropolitan Manila which correlates well with the findings from other population genetic studies of *Ae*. *aegypti* in a microgeographic-scale setting [19,21,22]. In a study conducted in Brazil, the level of genetic differentiation ranged from 0.002 to 0.094 with a maximum distance among collection sites of 30 km [22]. Urban cities in Southeast Asian countries with spatial scales of 5–50 km also showed low genetic differentiation (0.026–0.032) [19]. The same outcome (average *F*_ST_ = 0.037) was observed among villages in Thailand with geographical distances up to 27 km [21]. Our findings, along with those of previous studies, suggest that mosquito populations within fine-scale areas consist of similar allele frequencies, exhibiting continuous and active exchange or sharing of alleles. Moreover, our study revealed the lack of isolation-by-distance (IBD) which further implies that mosquito populations are not in equilibrium of migration and genetic drift at this spatial scale.

The lack of IBD and low *F*_ST_ imply high gene flow and weak genetic drift among mosquito populations in Metropolitan Manila. This high gene flow may indicate the possibility of “passive” dispersal of *Ae*. *aegypti* throughout the region. Other studies claim that this mosquito vector occasionally travels long distances by taking advantage of human-aided transportation routes via land, sea, or air [26,29,62]. This is evidenced by the eggs, larvae, and adults found in commercial trucks and ships through tire importation [25,63] while larvae and pupae are littered across transportation zones such as airports [64] and docks/ports [29,63]. Such a mechanism could facilitate high gene flow, resulting in genetically-similar mosquito populations. Additionally, spatial autocorrelation revealed limited dispersal distance up to 1 km, suggesting the individual flight range or “active dispersal” of *Ae*. *aegypti* [12]. The duality of both “passive” and “active” dispersal has been a common finding among human-adapted mosquitoes, especially in *Ae*. *aegypti* found in Southeast Asia [19,21,27, 65] and is considered a key factor in the persistence and resurgence of mosquito-borne diseases [66].

Furthermore, the low genetic differences due to weak genetic drift among mosquito populations are also reported. As evidenced by studies in Vietnam [20] and in the Philippines [28], genetic differences among mosquito populations tend to be lower during the rainy season compared to the dry season. These studies surmise that mosquito populations from the dry season recolonize breeding sites, leading to the expansion and proliferation of the mosquito vector. The large population size at this period can decrease genetic drift which leads to low genetic differences among mosquito populations. A similar phenomenon was observed in areas where insecticide is applied periodically. Mosquito populations in untreated areas facilitated high mosquito abundance and weakened genetic drift, resulting to decreased genetic differentiation among mosquito populations [20].

Our Bayesian analysis inferred sympatric multiple genetic clusters (K = 3 to K = 6) similar to the results reported by other population genetic studies with micro-geographic scales [19,22,67,68] and those conducted in the Philippines [28,29]. A possible explanation for this phenomenon could be the result of divergence from a single ancestry that produced multiple genetic clusters over time. It is argued that a single house or closely situated houses could act as an assembling unit in the formation of multiple genetic clusters in fine-scale areas [19,26,69]. Another possible explanation for multiple genetic clusters could be various invasions or introductions of *Ae*. *aegypti* populations from neighboring regions or provinces surrounding Metropolitan Manila. These has been shown in studies in China [70] and the US [71]. We attempted to test this hypothesis by obtaining publicly available genetic data from previous studies in the Philippines [17, 28, 29], however only two microsatellite loci (AC2 and AC4) matched with our set of markers. Furthermore, we expanded our search to neighboring Southeast Asian countries (e.g. Thailand, Vietnam and Indonesia) using the database vectorbase.org [44], but obtained the same outcome of matched loci (AC2 and AC4). This perspective is an interesting avenue for future investigations regarding population genetic studies of *Ae*. *aegypti* in the Philippines.

Although this analysis indicated multiple genetic clusters, mixed ancestry is likewise noticeable where each individual mosquito is assigned to different genetic clusters. The bar plots showed clear patterns of genetic admixture across mosquito populations. The interpretations of our Bayesian analysis are consistent with other population genetic studies [19,22,28,29,67,68] which demonstrated low genetic differentiation with no discernable genetic structures. The constant migration of mosquito populations facilitated by passive dispersal (e.g. human-mediated transportation) could have produced the genetic admixture mosquito populations seen in our results.

The constructed dendrogram allows us to further examine the passive dispersal of *Ae*. *aegypti* by describing potential dispersal patterns and its impact on the fine-scale genetic structuring. The first group (Fig 2, green line/circles) demonstrates the spanning network of genetically similar mosquito populations from the northern up to the southern parts of the region. The long-distance genetic similarity among these mosquito populations could be due to the major roads that connect the north and south. This supports the claim that long-distance dispersal of the mosquito vector could be facilitated through human-aided transportation.

Interestingly, the mosquito populations in the eastern (Fig 2, red line/circles) and northwestern (Fig 2, yellow line/circles) areas highlight the patterns of dispersal and features that could affect fine-scale genetic structuring. These areas have a unique landscape feature of being marginally separated from neighboring cities. For example, a large highway and river split a sizeable land area in cities of Marikina (MRK) and Pasig (PSG) while large highways (e.g., expressways) divide Valenzuela (VAL) and South Caloocan (CAL-S). As a result, only a few roads and bridges connect to these cities which limits access to neighboring areas. This circumstance may limit migration between mosquito populations, resulting in formation of distinct genetic groups as shown in the dendrogram (MRK & PSG and VAL & CAL-S). Landscape fragmentation can limit gene flow among mosquito populations as evidenced in the West Indies, producing defined mosquito populations on the east and west side of a major highway [72]. This could also explain the TGG mosquito population from Taguig City since the land area is separated by two large highways and a river which limits access to neighboring cities (Fig 2, black line/circle).

The genetic grouping shown in the dendrogram can also be explained by the occurrence and magnitude of flooding in selected cities within the region. From 2009 to 2014, Metropolitan Manila had experienced several flooding events which displaced residents and damaged properties and businesses [73]. Porio [74] identified the cities of Taguig, Pasig, Marikina, Valenzuela, and Caloocan as flood-prone areas, among many others. Because of these cities marginally separated, we hypothesize that, after a flooding event, alleles within these populations could be generally preserved and its allelic combination may become distinct from other mosquito populations over time due to limited gene flow.

Our findings along with those from previous reports in the Philippines [28,29] provide a partial picture of the current population genetic status of the mosquito vector, *Ae*. *aegypti*, in the country. The low genetic differentiation observed in all the reports including ours, indicates its widespread distribution not only within cities and villages but also in different islands of the country. Human-mediated transportation (marine or road transportation) facilitate the long dispersal capability of *Ae*. *aegypti*, enabling the observed admixture within the mosquito population. This comprehensive illustration of the population biology of *Ae*. *aegypti* in the Philippines strengthens our understanding for developing better vector control strategies. If and when the country pursues innovative vector control approaches, these findings will be integral to the success of the program.

## Supporting information

S1 Fig Isolation-by-distance of pairwise *F*_ST_ and geographic distance of *Ae*. *aegypti* populations in Metropolitan Manila, Philippines. (TIF)

S2 Fig Correlogram of spatial autocorrelation showing the coefficient (r) up to 45 km with 1-kilometer intervals. U and L are upper and lower confidence interval limit respectively. The point and whiskers plot for each distance class represent the 95% confidence intervals around the mean r values generated by bootstrapping (10,000 replicates).
(TIF)

S3 Fig Graph of ΔK against K showing the probable number of genetic clusters in (a) in all mosquito individuals [n = 526] in 21 study areas and (b-f) datasets of standardized mosquito individuals per study area [n = 210, 10 individuals per study area].
(TIF)

S1 Table *Ae aegypti* collection sites and population size in Metropolitan Manila, Philippines. (XLSX)

S2 Table List and characteristics of microsatellites markers in *Ae*. *aegypti* used in this study. (XLSX)

S3 Table Allele frequencies of the 11 microsatellite loci in *Ae aegypti* populations in Metropolitan Manila. (XLSX)

S4 Table Analysis of the genetic diversity of *Ae aegypti* using 11 microsatellite loci. (XLSX)

S5 Table Null allele frequency estimates per locus per *Ae aegypti* population. (XLSX)

S6 Table Analysis of linkage disequilibrium of *Ae*. *aegypti* populations in Metropolitan Manila, Philippines. (XLSX)

S7 Table Genetic differentiation of *Ae*. *aegypti* populations in Metropolitan Manila, Philippines using uncorrected (below diagonal) and corrected (above diagonal) *F*_ST_ values. (XLSX)

S8 Table Genetic differentiation of *Ae*. *aegypti* populations in Metropolitan Manila, Philippines using corrected *F*_ST_ (below diagonal) and gene flow (*Nm*) (above diagonal). (XLSX)

S9 Table Analysis of molecular variance (AMOVA) of populations from Metropolitan Manila. (XLSX)
