# Supplementary figures and images for: Fine-scale population genetic structure of dengue mosquito vector, Aedes aegypti, in Metropolitan Manila, Philippines
Source: PLoS Negl Trop Dis. 2020 May 4;14(5):e0008279. doi: 10.1371/journal.pntd.0008279 (PMC7224578; doi:10.1371/journal.pntd.0008279)

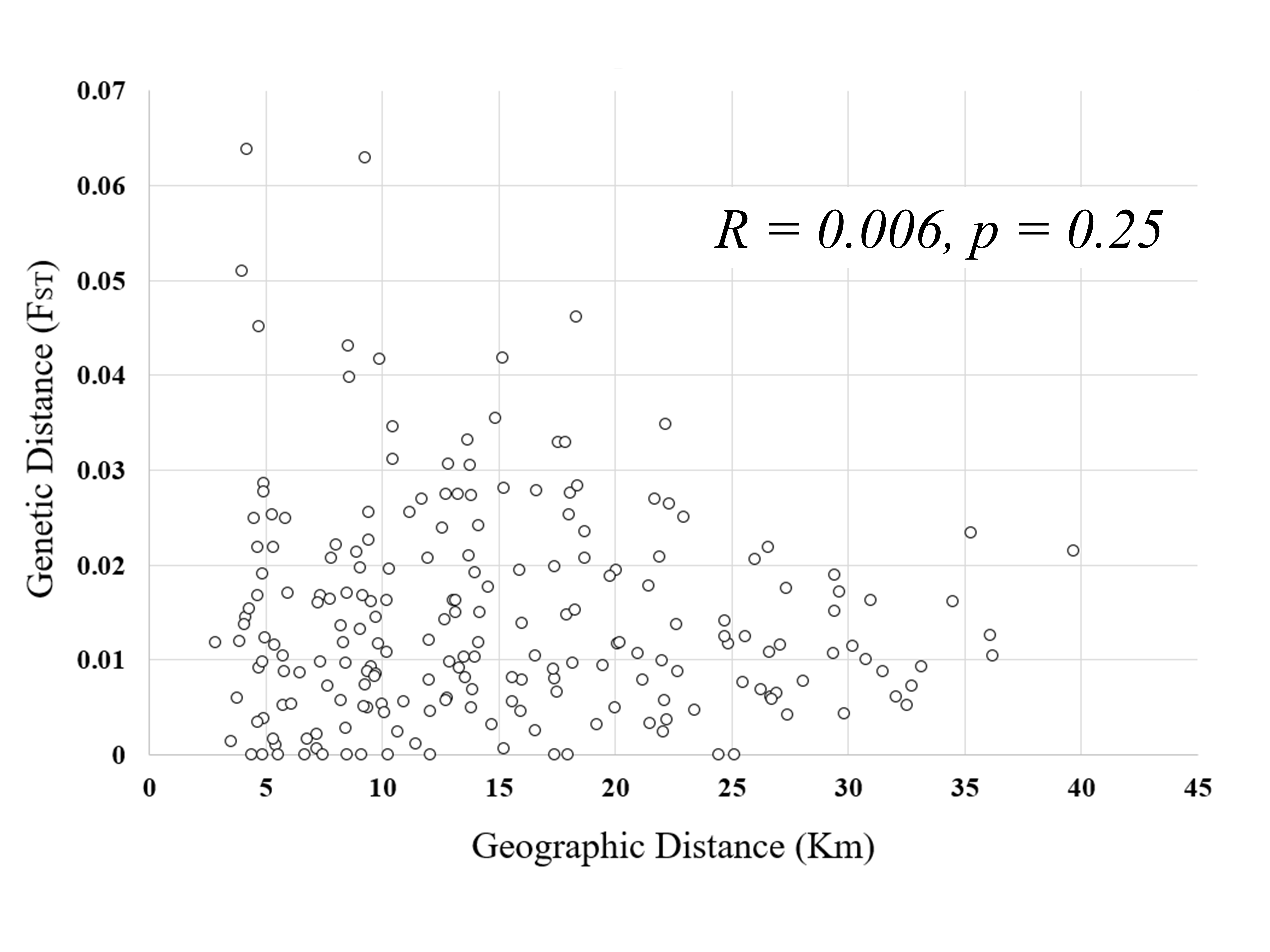

Supplement: S1 Fig — (TIF) [file pntd.0008279.s001.TIF]

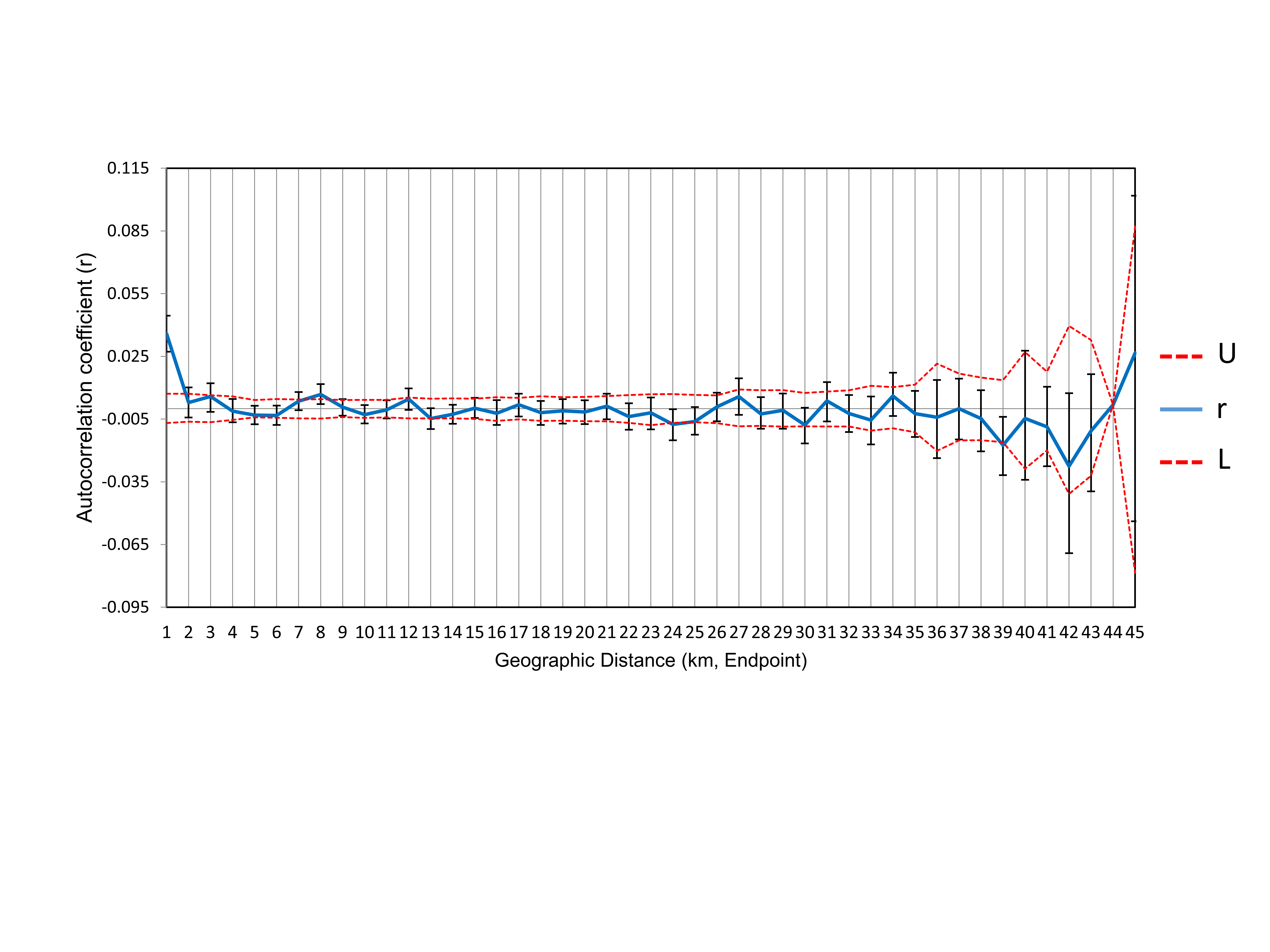

Supplement: S2 Fig — U and L are upper and lower confidence interval limit respectively. The point and whiskers plot for each distance class represent the 95% confidence intervals around the mean r values generated by bootstrapping (10,000 replicates). (TIF) [file pntd.0008279.s002.TIF]

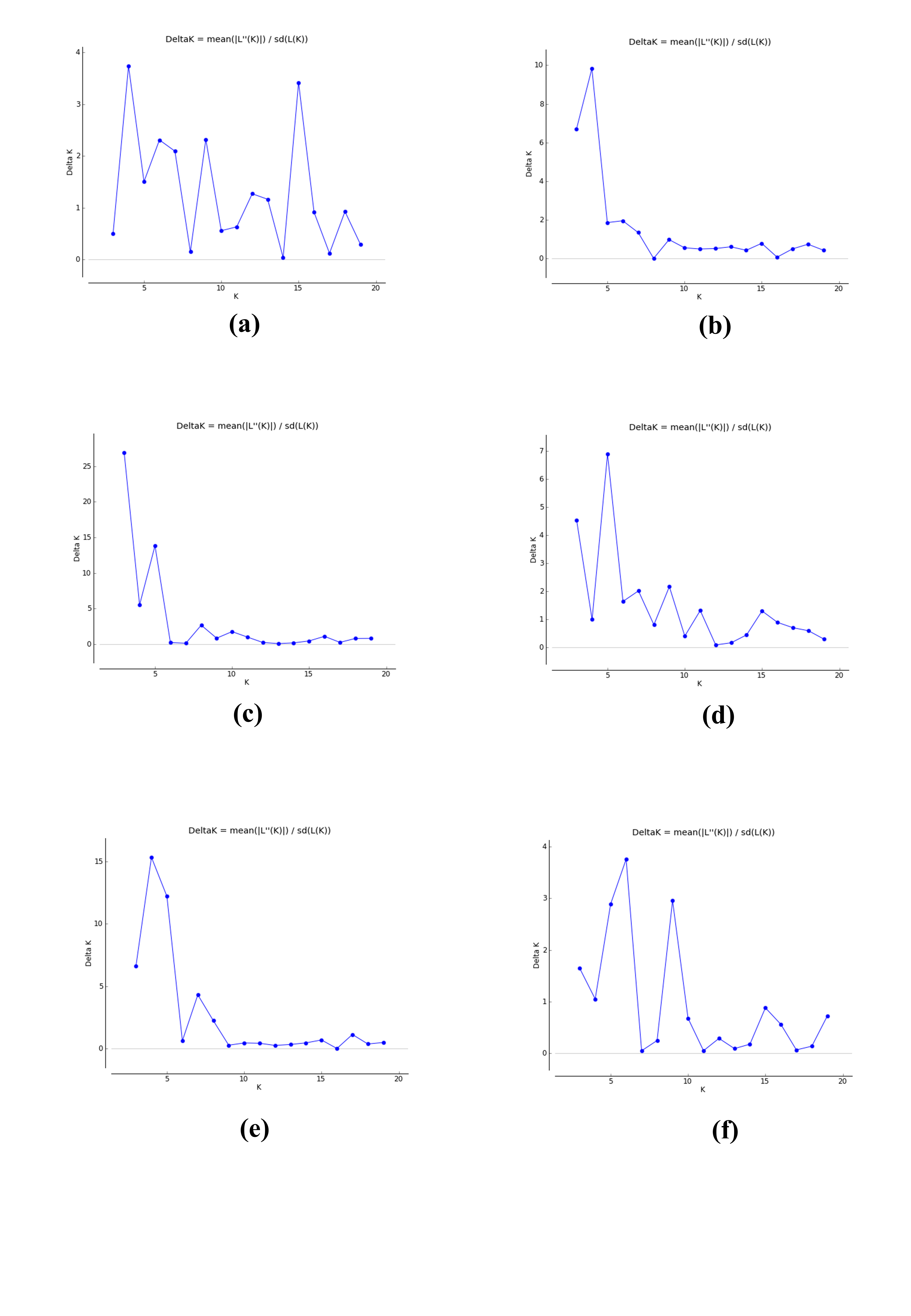

Supplement: S3 Fig — Graph of ΔK against K showing the probable number of genetic clusters in (a) in all mosquito individuals [n = 526] in 21 study areas and (b-f) datasets of standardized mosquito individuals per study area [n = 210, 10 individuals per study area]. (TIF) [file pntd.0008279.s003.tif]
